# Behandlung von Knochen- und Protheseninfektionen mit Bakteriophagen: Ein systematisches Review

**DOI:** 10.1007/s00132-021-04148-y

**Published:** 2021-09-09

**Authors:** Nike Walter, Li Deng, Christoph Brochhausen, Volker Alt, Markus Rupp

**Affiliations:** 1 Klinik und Poliklinik für Unfallchirurgie, Universitätsklinikum Regensburg, Franz-Josef-Strauß-Allee 11, 93053 Regensburg, Deutschland; 2 Abteilung für Psychosomatische Medizin, Universitätsklinikum Regensburg, Regensburg, Deutschland; 3 Institut für Virologie, Helmholtz Zentrum, München, Deutschland; 4 Institut für Pathologie, Universitätsklinikum Regensburg, Regensburg, Deutschland

**Keywords:** Alternative Therapie, Antibiotikaresistenz, Phagentherapie, Protheseninfektion, Behandlungs-Outcome, Alternative therapy, Antibiotic resistance, Phage therapy, Periprosthetic infection, Treatment outcome

## Abstract

**Hintergrund:**

Die Behandlung von Knochen- und Protheseninfektionen bleibt trotz moderner Behandlungskonzepte mit interdisziplinärem Therapieansatz schwierig und weitere Maßnahmen zur Verbesserung des Behandlungsergebnisses sind wünschenswert. Präklinischen Studien liefern ein vielversprechendes Bild der Wirksamkeit von Bakteriophagen zur Behandlung von Knochen- und Protheseninfektionen.

**Ziel der Arbeit:**

Die vorliegende Arbeit gibt eine systematische Übersicht über die klinische Anwendung von Bakteriophagen zur Behandlung von Knochen- und Protheseninfektionen.

**Material und Methoden:**

Eine systematische Suche wurde in PubMed zur Identifikation von primären klinischen Daten zur Anwendung der Phagentherapie bei Patienten mit Knochen- und Protheseninfektion durchgeführt.

**Ergebnisse:**

Elf Studien wurden eingeschlossen, bestehend aus 8 Fallberichten und 3 Fallserien. Indikationen der Phagentherapie waren periprothetische Infektionen (*n* = 12, 52,2 %), frakturassoziierte Infektionen (*n* = 9, 39,1 %), Osteomyelitis (*n* = 1, 4,4 %) und eine Iliosakralgelenkinfektion nach Zementaugmentation einer Metastase (*n* = 1, 4,4 %). Die Interventionen waren heterogen, Phagen wurden intravenös verabreicht, intraoperativ ins Gelenk injiziert, intraoperativ lokal angewendet oder über Drainagen appliziert. In Kombination mit Antibiotikatherapie konnte eine vollständige Infekteradikation bei 18 Patienten (78,3 %) erreicht werden. Bei 91,3 % der Patienten wurden keine Nebenwirkungen berichtet.

**Schlussfolgerung:**

Bakteriophagen sind eine vielversprechende Behandlungsmethode von Knochen- und Protheseninfektionen in Kombination mit einer Antibiotikatherapie. Zukünftige klinische Studien mit höherem Evidenzgrad werden benötigt, um eine erfolgreiche Translation der Bakteriophagentherapie in die klinische Praxis weiter zu etablieren.

## Abstract (en)

**Background:**

The treatment of bone and prosthesis infections remains difficult despite modern treatment concepts. Further tools for the improvement of treatment outcome are desirable. Preclinical studies provide promising evidence of the efficacy of bacteriophages for the treatment of bone and joint infections.

**Objectives:**

The present work provides a systematic review of the clinical application of bacteriophages for the treatment of bone and joint infections.

**Materials and methods:**

A systematic search was performed in PubMed to identify primary clinical data on the use of phage therapy in patients with bone and joint infection.

**Results:**

Eight case reports and three case series were included in the study. Indications for phage therapy were periprosthetic joint infection (*n* = 12, 52.2%), fracture-related infection (*n* = 9, 39.1%), osteomyelitis (*n* = 1, 4.4%) and sacroiliac joint infection after cement augmentation of a metastasis (*n* = 1, 4.4%). Interventions were heterogeneous; phages were administered intravenously, injected intraoperatively into the joint, applied locally intraoperatively, or administered via drains. In combination with antibiotic therapy, complete infection eradication was achieved in 18 patients (78.3%). No side effects were reported in 91.3% of patients.

**Conclusion:**

Bacteriophages represent a promising treatment option for bone and prosthesis infections in combination with antibiotic therapy. Future clinical trials with a higher level of evidence are required for the successful translation of bacteriophage therapy into clinical practice.

## Hinführung zum Thema

Infektionen nach endoprothetischem Gelenkersatz oder Frakturosteosynthese sind eine Herausforderung in Orthopädie und Unfallchirurgie. Vor dem Hintergrund zunehmender Antibiotikaresistenzen rücken Behandlungsalternativen zur etablierten Antibiotikatherapie in den Fokus. Insbesondere die Anwendung von Bakteriophagen scheint laut präklinischen Studien Wirksamkeit bei implantatassoziierten Infektionen zu besitzen. In diesem Beitrag wird eine Übersicht über primäre klinische Daten zur Anwendung der Bakteriophagentherapie bei Patienten mit Knochen- und Protheseninfektionen vorgestellt.

## Hintergrund und Fragestellung

**Figure 1:**
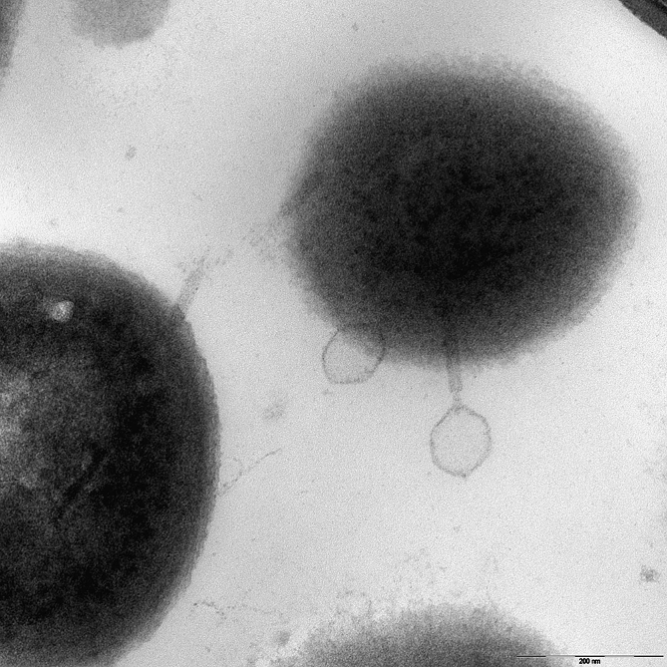

Der endoprothetische Gelenkersatz und die operative Frakturversorgung sind lebensverbessernde operative Verfahren für Millionen von Menschen auf der ganzen Welt. Neben den funktionellen Vorteilen, die eine Implantation von Endoprothesen oder Osteosynthesematerial bewirken können, besteht für jedes Implantat das Risiko einer Implantatinfektion. Ein Schlüsselelement in der Pathophysiologie von implantatassoziierten Knocheninfektionen ist die bakterielle Kolonisierung des Implantats und anschließende Biofilmbildung [29]. Schon frühe Studien identifizierten das sogenannte „Race for the surface“-Phänomen, einen Wettstreit zwischen körpereigenen Zellen und Bakterien um die neue Besiedlungsoberfläche [14, 15]. Gerade die Infekteradikation nach Bildung eines chronischen Biofilms stellt eine besondere klinische Herausforderung dar. Die Diffusion von antimikrobiellen Wirkstoffen in Biofilme wird durch mehrere Faktoren wie die physikalische Barriere, den verstärkten Austausch von Antibiotikaresistenzgenen und langsamere Wachstumsraten deutlich eingeschränkt [1]. Infolgedessen sind Biofilmmikroorganismen bis zu 1000-mal resistenter gegen wachstumsabhängige antimikrobielle Wirkstoffe als ihr planktonisches Äquivalent [26, 30]. Entsprechend hängen Behandlungskonzepte für Knochen- und Protheseninfektionen hauptsächlich von der Dauer der Infektion ab, wobei der Reifungszustand des Biofilms als ein Hauptfaktor für die therapeutische Möglichkeit des Implantaterhaltes angesehen wird [36]. Gerade vor dem Hintergrund zunehmender Antibiotikaresistenzen wird nach Alternativen zur etablierten Antibiotikatherapie gesucht. Ein vielversprechender Ansatz sind Bakteriophagen, die ubiquitär in der Umwelt auftreten und zu den am häufigsten vorkommenden biologischen Organismen zählen (Abb. 1; [5]). Diese Viren infizieren Bakterien selektiv, replizieren sich und werden schließlich durch Lyse freigesetzt, wodurch der Wirt getötet wird.

Die Bakteriophagentherapie ist keine neue Behandlungsmethode. Felix d’Herelle hatte die Möglichkeit der zuvor vom Briten Twort im *The Lancet* beschriebenen Bakterienlyse für die klinische Anwendung erkannt und letztlich Shigellen-Enteritis bei französischen Truppen im 1. Weltkrieg erfolgreich behandelt. Er war es auch, der den Begriff „Bakteriophage“ geprägt und aufgrund seiner Errungenschaften 1925 mit der Leeuwenhoek-Medaille ausgezeichnet wurde [4, 6]. Durch die Entdeckung des Penicillins war der Behandlungsansatz mit Bakteriophagen insbesondere in der westlichen Welt nach dem 2. Weltkrieg mehr oder weniger in Vergessenheit geraten. Indes wurden Bakteriophagen in der ehemaligen Sowjetunion zur Therapie verschiedener Infektionen, wie gastrointestinalen Infekten aber auch Gasbrand bei Soldaten, angewandt. Trotz der dortigen breiten Anwendung, sind nur wenige Publikationen in englischer Sprache verfügbar [27]. Ein Beispiel sind Veröffentlichungen des Instituts für Immunologie und Experimentelle Therapie, auch bekannt als „Hirszfeld Institut“ im heutigen Polen, wo zwischen 1981 und 1999 1857 Fälle mit Bakteriophagen behandelt und 85–92,4 % erfolgreiche Ergebnisse berichtet wurden [25, 33].

Die zunehmende Ausbreitung der antimikrobiellen Resistenz, zusammen mit der Entwicklung von Analysetechniken wie Hochdurchsatzsequenzierungen und Elektronenmikroskopie, die es ermöglichen Phagen genauer zu untersuchen, führten zu einer Renaissance der Phagentherapie [2]. Jedoch ist bis heute die Bakteriophagentherapie in Deutschland nur im Einzelfall als Heilversuch anwendbar. Aktuell gibt es kein zugelassenes Bakteriophagenprodukt zur klinischen Anwendung in der EU.

Präklinische Studien weisen darauf hin, dass die Phagentherapie das Potenzial besitzt, die Behandlung von knochen- bzw. implantatassoziierten Infektionen zu verbessern und zeigen Vorteile von Phagen gegenüber Antibiotika, wie Wirtsspezifität und geringe Toxizität für den Menschen auf [13]. Daher soll in diesem systematischen Review eine Übersicht bisheriger Entwicklungen in Bezug auf die klinische Anwendung von Bakteriophagen gegeben werden. Insbesondere soll dabei der Evidenzgrad der klinischen Studien, sowie die Sicherheit und Wirksamkeit der Phagentherapie evaluiert werden.

## Studiendesign und Untersuchungsmethoden

Die elektronische Datenbank PubMed wurde durchsucht. Die Suche wurde mit den folgenden Begriffen durchgeführt: („Bacteriophage*“ OR „phage*“) AND („periprosthetic*“ OR „joint infection*“ OR „implant-associated infection*“ OR „fracture-related infection“ OR „bone infection*“ OR „osteomyelitis*“OR „musculoskeletal*“). Alle Artikel wurden anhand der Titel und Zusammenfassungen darauf überprüft, ob primäre klinische Daten zur Anwendung der Phagentherapie bei Patienten mit Knochen- und Protheseninfektion berichtet werden. Studien, die nicht in englischer oder deutscher Sprache verfügbar waren, vor dem Jahr 2000 durchgeführt wurden oder Fälle mit Haut- und Weichteilinfektionen beinhalteten, wurden ausgeschlossen. Ebenfalls fanden Studien, die über die Verwendung von aus Phagen gewonnenen Produkten (z. B. Endolysine) berichten und nicht „peer-review“ publiziert wurden, keine Berücksichtigung. Zusätzlich wurden Primärquellen von Übersichtsarbeiten analysiert. Die Auswahl der Studien wurde unabhängig voneinander von zwei Autoren (NW, MR) durchgeführt. Diskrepanzen wurden einvernehmlich geklärt. Diese Arbeit wurde in Übereinstimmung mit den PRISMA(Prefered Reporting Items for Systematic Reviews and Meta-Analysis)-Richtlinien durchgeführt [20]. Da es sich um eine reine Literaturstudie handelt und dieser Beitrag keine Studien an Menschen oder Tieren beinhaltet, wurde auf ein Ethikvotum verzichtet.

## Ergebnisse

**Figure 2:**
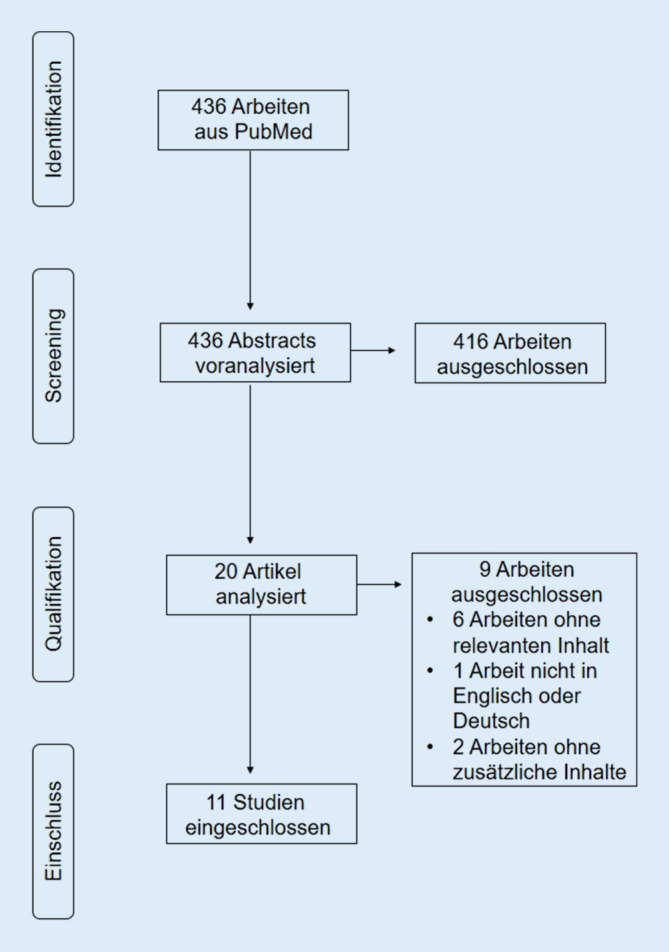

Die systematische Suche ergab 436 Treffer. Nach Anwendung der Ausschlusskriterien verblieben 20 Studien, die anhand des Volltextes überprüft wurden. Hiervon wurden 9 weitere ausgeschlossen, *n* = 6 aufgrund nicht relevanter Daten für die Studie, *n* = 1 aufgrund keiner Verfügbarkeit in englischer Sprache und *n* = 2, die Daten beinhalteten, die bereits in einer eingeschlossenen Publikation berichtet wurden. Insgesamt wurden 11 Studien in die Übersichtsarbeit eingeschlossen (Tab. 1; Abb. 2; [3, 8–12, 21–23, 28, 32]).

**Table 1:** 

Autoren, Jahr, Ort	Indikation, Stichprobengröße, Alter und Geschlecht	Pathogene	Phagentyp und Sensitivität	Intervention	Phagenkonzentration	Ergebnisse	Nebenwirkungen
Cano et al. 2020, USA, [3]	PJI, *n* = 1; 62 Jahre, m	*Klebsiella pneumoniae*	KpJH46φ (Adaptive Phage Therapeutics [APT], Gaithersburg, MD, USA)	Intravenöse Anwendung, zusätzlich Antibiotikatherapie	40 tägliche Infusionen 50 ml (6 × 10^10^ PFU) in 0,9 % Kochsalzlösung	Infekteradikation Follow Up: 8,5 Monate	Keine berichtet
Doub et al. 2020, USA, [7]	PJI, *n* = 1; 72 Jahre, m	*S. aureus* (MRSA)	Anti-Staphylococci-Phage SaGR51φ (Adaptive Phage Therapeutics), Sensitivität extern bestätigt	Intraartikuläre Anwendung und tägliche intravenöse Gabe, zusätzlich Antibiotikatherapie	Intraartikulär zwei Dosen 5,4 × 10^9^ PFU in 10 ml Kochsalzlösung; intravenös 2,7 × 10^9^ PFU in 50 ml Kochsalzlösung	Infekteradikation Follow Up: 8 Monate	Nach dritter intravenöser Dosis erhöhte Werte von Aspartat-Aminotransferase, Alanin-Aminotransferase und Abbruch der intravenösen Bakteriophagentherapie
Ferry et al. 2020, Frankreich, [11]	PJI, *n* = 1; 49 Jahre, m	*S. aureus* (MSSA)	Zwei Anti-Staphylococci-Phagen (PP1493 und PP1815), (Pherecydes Pharma, Nantes, Frankreich), Sensitivität bestätigt	Lokale Anwendung von DAC® Hydrogel als Phagenträger, zusätzlich Antibiotikatherapie	300 mg steriles DAC®-Pulver gefüllt mit 1 ml jeder Bakteriophage (10^10^ PFU/ml) und 5 ml sterilem Wasser	Amputation	Keine berichtet
Ferry et al. 2020, Frankreich, [10]	PJI, *n* = 3; 80 Jahre, m; 84 Jahre, m; 83 Jahre, w	*S. aureus* (MSSA)	Drei Anti-Staphylococci-Phagen (PP1493, PP1815, PP1957), (Pherecydes Pharma), Sensitivität bestätigt	Intraoperative Injektion ins Gelenk, zusätzliche Antibiotikatherapie	Einmalig 1 × 10^9^ PFU/ml	Infekteradikation in zwei Patienten. Follow-Up: 7 Monate und 2,5 Jahre. Bestehende Fistel bei *n* = 1. Follow Up: 11 Monate	Keine berichtet
Tkhilaishvili et al. 2020, Deutschland, [27]	PJI, *n* = 1; 80 Jahre, w	*Pseudomonas aeruginosa*	Anti-Pseudomonas-Phage, (Eliava Institute, Tiflis, Georgien), Sensitivität bestätigt	Lokale Anwendung intraoperativ und wiederholt über Drainagen, zusätzliche Antibiotikatherapie	Lokal 100 ml (10^9^ PFU/ml), dann 5 ml (10^8^ PFU/ml) alle 8 h über 5 Tage	Infekteradikation. Follow Up: 10 Monate	Keine berichtet
Onsea et al. 2019, Belgien, [21]	FRI, *n* = 4; Alter und Geschlecht nicht berichtet	*S. epidermidis* und *Pseudomonas aeruginosa* (*n* = 2), *S. aureus* und *Streptococcus agalactiae* (*n* = 1), *Enterococcus faecalis* (*n* = 1)	BFC1-Cocktail (Queen Astrid Military Hospital, Brüssel, Belgien) und „Pyo-Phage“-Cocktail (Eliava Institute), Sensitivität bestätigt	Lokale, intraoperative Spülung, Implantation eines Gentamicin-imprägnierter Kollagenschwamm getränkt in Phagenlösung, postoperativ wiederholte Anwendung über Drainage, zusätzlich Antibiotikatherapie	Lokal 10–40 ml (10^7^ PFU/ml in 0,9 % Kochsalzlösung)	Infekteradikation in allen Fällen. Follow Up: 8–16 Monate	Ein Patient zeigte lokale Rötung und Schmerzen während der Administration des „Pyo-Phage“-Cocktails über die Drainage
					Postoperativ 3‑mal täglich für 7–10 Tage, mit individuellen Protokollen		
Nir-Paz et al. 2019, Israel, [20]	FRI, *n* = 1; 42 Jahre, m	*Acinetobacter baumannii,* *Klebsiella pneumoniae*	Phagen AbKT21φ3 und KpKT21φ1 (US-Naval Medical Research Centre Phage Bank), Sensitivität bestätigt	Wiederholte intravenöse Verabreichung, zusätzlich Antibiotikatherapie	1 ml jeder Phage (Titer nicht berichtet) für 35 min über 5 Tage. Nach einer Woche nochmals 1 ml der Anti‑A.-baumannii-Phage über 6 Tage	Infekteradikation. Follow Up: 8 Monate	Keine berichtet
Ferry et al. 2018, Frankreich, [9]	PJI, *n* = 1; 80 Jahre, w	*Pseudomonas aeruginosa, S. aureus*	Zwei individualisierte Phagencocktails (jeweils drei Phagen) (Pherecydes Pharma), Sensitivität für Anti-Pseudomonas-Phage und 2/3 Anti-Staphylococcus-Phagen bestätigt	Intraoperative Injektion ins Gelenk, zusätzliche Antibiotikatherapie	Einmalig 6 ml jedes Phagen-Cocktails (10^10^ PFU/ml) in jeweils 10 ml Kochsalzlösung	Infekteradikation. Follow Up 18 Monate	Keine berichtet
Ferry et al. 2018, Frankreich, [8]	Infektion des Iliosakralgelenks nach Zementaugmentation in Folge einer Chemotherapie bei Lungenkarzinom, *n* = 1; Anfang 60, m	*Pseudomonas aeruginosa*	Individualisierter Phagen-Cocktail (4 Phagen) (Pherecydes Pharma), Sensitivität bestätigt	Lokale, intraoperative Anwendung, lokale Wundanwendung, zusätzliche Antibiotikatherapie (lokal und intravenös)	1,2–9,7 × 10^8^ PFU/ml in 30 ml Kochsalzlösung	Infekteradikation, Patient verstarb am Lungenkarzinom an Tag 45 postoperativ	Keine berichtet
Patey et al. 2018, Frankreich, [22]	FRI, *n* = 4; 44 Jahre, m; 68 Jahre, w; 25 Jahre, m; 40 Jahre, w	*S. aureus* (*n* = 6)	„Pyo-Phage“-Cocktail (Eliava Institute und Microgen, Moskau, Russland)	Postoperative Anwendung, nicht weiter spezifiziert, zusätzliche Antibiotikatherapie	Nicht berichtet	Infekteradikation in 6/8 Fällen	Keine berichtet
	Osteomyelitis, *n* = 1; 84 Jahre, m	*S. aureus* und *Pseudomonas aeruginosa* (*n* = 1)					
	PJI, *n* = 3; 72 Jahre, w; 80 Jahre, w	*Pseudomonas aeruginosa* (*n* = 1)				Keimwechsel in zwei Fällen	
Vogt et al. 2017, Deutschland, [31]	PJI, *n* = 1; 33 Jahre, m	*Pseudomonas aeruginosa*, *Acinetobacter baumannii*	„Pyo-Phage“-Cocktail (Eliava Institute), Sensitivität bestätigt	Wiederholte Injektion „Pyo-Phage“-Cocktail über Drainagen	Nicht berichtet	Amputation	Keine berichtet

*FRI* frakturassoziierte Infektion („fracture-related infection“), *MRSA* Methicillin-resistenter *Staphylococcus aureus*, *MSSA* Methicillin-sensitiver *Staphylococcus aureus*, *PFU* „plaque forming units“, *PJI* periprothetische Gelenkinfektion („periprosthetic joint infection“), *w* weiblich, *m* männlich

Durchgeführt wurden die Studien in Frankreich (*n* = 5), den Vereinigten Staaten (*n* = 2), Deutschland (*n* = 2), Israel (*n* = 1) und Belgien (*n* = 1). Identifiziert wurden *n* = 8 Fallberichte und *n* = 3 Fallserien. Insgesamt wurden die Daten von 23 Patienten berichtet. Indikationen der Phagentherapie waren periprothetische Infektionen (*n* = 12, 52,2 %), frakturassoziierte Infektionen (*n* = 9, 39,1 %), Osteomyelitis (*n* = 1, 4,4 %) und eine Iliosakralgelenkinfektion nach Zementaugmentation einer Metastase (*n* = 1, 4,4 %). Die zugrundeliegende Infektion war monobakteriell in 16 der Fälle (69,6 %) und polybakteriell in 7 Fällen (30,4 %). Die häufigsten Erreger waren *Staphylococcus aureus* (*n* = 14, 46,7 %) und *Pseudomonas aeruginosa *(*n* = 8, 26,7 %). Ein vorheriger Sensitivitätstest der Phagen hinsichtlich der zugrundeliegenden Keime wurde in 9/11 Artikeln berichtet. Die Phagen wurden intravenös verabreicht, intraoperativ ins Gelenk injiziert, intraoperativ lokal angewendet oder über Drainagen appliziert. Zehn von elf Studien (90,9 %) berichten eine ergänzende Antibiotikatherapie. Keine der Studien erlaubte eine Unterscheidung zwischen Effekten durch die Phagen und denen der Antibiotikatherapie. In neun Studien wurden keine Nebenwirkungen der Phagentherapie berichtet (Patienten: *n* = 21; 91,3 %). Ein Patient zeigte eine lokale Rötung und Schmerzen während der Administration des Phagencocktails über die Drainage [22]. Bei einem weiteren traten nach dritter intravenöser Dosis erhöhte Leberwerte (Aspartat-Aminotransferase und Alanin-Aminotransferase) auf, woraufhin die intravenöse Bakteriophagentherapie abgebrochen wurde [8]. Eine vollständige Infekteradikation über mindestens 7 Monate konnte bei 18 Patienten (78,3 %) erreicht werden. Bei zwei Fällen erfolgte ein Keimwechsel [23] und bei einem Patienten blieb eine Fistel bestehen [11]. In zwei Fallstudien erfolgte kein Extremitätenerhalt durch die Phagentherapie [12, 32].

## Diskussion

Diese Arbeit gibt eine systematische Übersicht über den klinischen Einsatz der Phagentherapie bei Knochen- und Protheseninfektionen. Insgesamt wurden seit dem Jahr 2000 23 Fälle mit einer Knochen- oder Protheseninfektion berichtet, die mit Bakteriophagen therapiert wurden. Bei 78,3 % der 22 Patienten konnte eine vollständige Infekteradikation für mindestens 7 Monate erreicht werden. In 91,3 % der Fälle konnten keine Nebenwirkungen der Bakteriophagentherapie beobachtet werden.

In Anbetracht der steigenden Antibiotikamultiresistenz erscheint der Ansatz, sich die natürlichen Feinde der Bakterien – nämlich die Bakteriophagen – therapeutisch zu Nutze zu machen, naheliegend und erfolgsversprechend [19]. Eine beträchtliche Anzahl von 11,6- 22,6 % der periprothetischen Gelenkinfektionen werden durch sogenannte „Difficult-to-treat“(DTT)-Pathogene verursacht, die resistent gegenüber biofilmaktiven Antibiotika sind [31, 37]. DTT-Infektionen sind mit einer erhöhten Mortalität verbunden, führen zu schlechteren klinischen Ergebnissen und sind mit höheren Revisionsraten vergesellschaftet [31, 35]. Auch polymikrobielle Infektionen verkomplizieren den Behandlungsverlauf periprothetischer Infektionen [34]. Hier erscheint es zielführend, Phagen in Kombination mit Antibiotikatherapie anzuwenden.

Neben dem therapeutischen Nutzen, welcher durch eine additive Bakteriophagentherapie in präklinischen Studien impliziert wird, könnten Phagen auch zur Prävention implantatassoziierter Infektionen eingesetzt werden [24]. Hier bestehen diverse Möglichkeiten der Beladung von Materialien wie Keramik, K‑Drähte [17] oder Hydrogel [18]. Jedoch waren die identifizierten Studien gering in ihrer Anzahl und zeigten eine Inhomogenität sowohl bezüglich (1) der Indikation, (2) der Pathogene und (3) der Art der Anwendung. Letztere erfolgte intravenös, intraartikulär, lokal oder über Drainagen mit unterschiedlicher Dauer. Dabei war die Dosis, der Zeitpunkt und die Häufigkeit der Verabreichung heterogen in der Literatur. Da Bakteriophagen in der EU nicht zur klinischen Anwendung zugelassen sind, handelt es sich bei den 11 identifizierten Studien um individuelle Heilversuche, aus welchen die optimale Behandlung mit Phagen nicht ableitbar ist. Zusätzlich unterschied keine der Studien zwischen Effekten durch die Phagen und denen der Antibiotikatherapie.

Für einen nächsten Schritt in Richtung effektiver Anwendung der Phagentherapie ist somit die Etablierung von vereinheitlichten Behandlungsprotokollen und internationalen Richtlinien unerlässlich [7]. Bei der systematischen Suche konnte keine randomisierte kontrollierte Studie identifiziert werden, welche die genannten Punkte adressiert. Derzeit ist lediglich eine klinische Studie zur Evaluierung der Sicherheit der Phagentherapie bei Knochen- und Protheseninfektionen registriert (https://clinicaltrials.gov/, Juni 2021). Um die Möglichkeiten der Bakteriophagentherapie zu evaluieren werden zukünftige klinische Studien mit höherem Evidenzgrad benötigt. Zusätzlich ist die routinemäßige und zeitnahe Gewinnung von Phagen und die Herstellung der Phagencocktails unter „Good Manufacturing Practice“-Bedingungen als Herausforderung zu betrachten. Zudem wird ein großer Teil der Erkenntnisse zur Phagentherapie von einer sehr begrenzten Anzahl von Phagenisolaten abgeleitet, was die weltweite Akzeptanz von Bakteriophagen als Therapeutika verlangsamt. Von den 10^31^ existierenden Phagen, wurden bisher weniger als 10^4^ isoliert und sequenziert (https://www.ncbi.nlm.nih.gov/, Juni 2021), weshalb die Weiterentwicklung von kulturunabhängigen Methoden in den Fokus gerückt werden sollte [16].

## Fazit für die Praxis

- In Anbetracht der steigenden Antibiotikamultiresistenz werden alternative Therapieansätze benötigt.
- Bakteriophagen sind eine vielversprechende Behandlungsmethode von Knochen- und Gelenksinfektionen in Kombination mit Antibiotikatherapie.
- Die Etablierung von optimalen Behandlungsprotokollen ist erforderlich.
- Zukünftige klinische Studien mit höherem Evidenzgrad werden für eine erfolgreiche Translation der Bakteriophagentherapie in die klinische Praxis benötigt.

## Abkürzungen

DTT: „Difficult-to-treat“
FRI: Frakturassoziierte Infektion („fracture-related infection“)
MRSA: Methicillin-resistenter *Staphylococcus aureus*
MSSA: Methicillin-sensitiver *Staphylococcus aureus*
PFU: „Plaque forming units“
PJI: Periprothetische Gelenkinfektion („periprosthetic joint infection“)
PRISMA: Prefered Reporting Items for Systematic Reviews and Meta-Analysis
